# White matter microstructural alterations in obstructive sleep apnea assessed by time-dependent diffusion MRI

**DOI:** 10.1007/s11604-026-01991-x

**Published:** 2026-04-16

**Authors:** Toshiaki Taoka, Rintaro Ito, Kunihiro Iwamoto, Seiko Miyata, Rei Nakamichi, Toshiki Nakane, Mami Iima, Hiroshige Fujishiro, Masashi Ikeda, Kazushige Ichikawa, Akifumi Kamiunten, Nobuyasu Ichinose, Yoshiki Tanaka, Shinji Naganawa

**Affiliations:** 1 Department of Innovative Biomedical Visualization (iBMV), Nagoya University, 65 Tsurumai-cho, Showa-ku, Nagoya, Aichi 466-8550 Japan; 2 Department of Radiology, Nagoya University, Nagoya, Japan; 3 Department of Psychiatry, Nagoya University, Nagoya, Japan; 4 Department of Fundamental Development for Advanced Low Invasive Diagnostic Imaging, Nagoya University, Nagoya, Japan; 5 Canon Medical Systems Corporation, Otawara, Japan; 6 SORD Corporation, Chiba, Japan

**Keywords:** Obstructive sleep apnea, Time-dependent diffusion MRI, White matter microstructure, Glymphatic system, DTI-ALPS

## Abstract

**Purpose:**

Diffusion-based neurofluid imaging indices, such as the DTI-ALPS method, are influenced by underlying white matter microstructure. This study aimed to examine associations between obstructive sleep apnea (OSA) severity and white matter microstructure using the difference in apparent diffusion coefficients between oscillating gradient spin-echo and pulsed gradient spin-echo sequences (ΔOGSE–PGSE), and to clarify the structural background influencing diffusion-based neurofluid imaging indices.

**Materials and methods:**

Forty patients with OSA underwent overnight polysomnography and time-dependent diffusion MRI. ΔOGSE–PGSE was calculated from OGSE- and PGSE-derived apparent diffusion coefficients and normalized to MNI standard space. Atlas-based region-of-interest (ROI) analysis was performed. Associations between ΔOGSE–PGSE and polysomnographic indices, including the apnea–hypopnea index (AHI) and hypopnea index (HI), as well as neurofluid-related imaging metrics—relative choroid plexus volume (rCPV), relative white matter hyperintensity volume (rWMHV), and the ALPS index—were evaluated using linear mixed-effects models. Voxel-based and ternary plot analyses were also performed.

**Results:**

ROI analysis demonstrated significant negative associations between ΔOGSE–PGSE and AHI across multiple regions, including white matter and subcortical structures. Exploratory analyses additionally identified associations involving HI and several cortical regions, including the insular and temporal cortices. Among neurofluid-related metrics, rCPV showed significant associations with ΔOGSE–PGSE, whereas no significant associations were observed for the ALPS index or rWMHV. Sensitivity analyses using ROI erosion and cluster-robust standard errors yielded consistent association patterns.

**Conclusion:**

ΔOGSE–PGSE demonstrated associations with white matter microstructural alterations related to OSA. These findings show that ΔOGSE–PGSE is sensitive to microstructural features not fully captured by conventional diffusion MRI. Although ΔOGSE–PGSE does not directly assess glymphatic function, it provides insight into shared microstructural features that form part of the structural background influencing diffusion-based neurofluid imaging indices, including ALPS, and supports their interpretation in obstructive sleep apnea.

**Supplementary Information:**

The online version contains supplementary material available at 10.1007/s11604-026-01991-x.

## Introduction

The brain glymphatic system has gained increasing attention as a mechanism involved in waste clearance and metabolic regulation [1]. As a noninvasive approach originally proposed as a proxy for glymphatic function, the diffusion MRI–based analysis along the perivascular space (ALPS) index was proposed [2] and has since been applied to a wide range of conditions, including aging, dementia, and sleep-related disorders [3–13]. Although the DTI-ALPS method is expected to indirectly reflect glymphatic function by capturing anisotropic diffusion along perivascular spaces, accumulating evidence indicates that it is also influenced by surrounding white matter microstructure [14–17]. Accordingly, interpretation of the DTI-ALPS method requires consideration of its structural background.

In the first phase of the MOONLIGHT (Multimodal Observation of Neurofluid through Imaging-based Glymphatic Transport) study, we demonstrated significant associations between the ALPS index and diffusion tensor imaging (DTI)–derived white matter metrics, including fractional anisotropy and mean diffusivity [11]. These findings suggested that the ALPS index may be influenced by white matter features emphasized by DTI, such as fiber orientation and tissue organization. This structural dependency has been further discussed in recent reviews, which have proposed interpretative frameworks incorporating white matter microstructural influences [18–20].

**Fig. 1:**
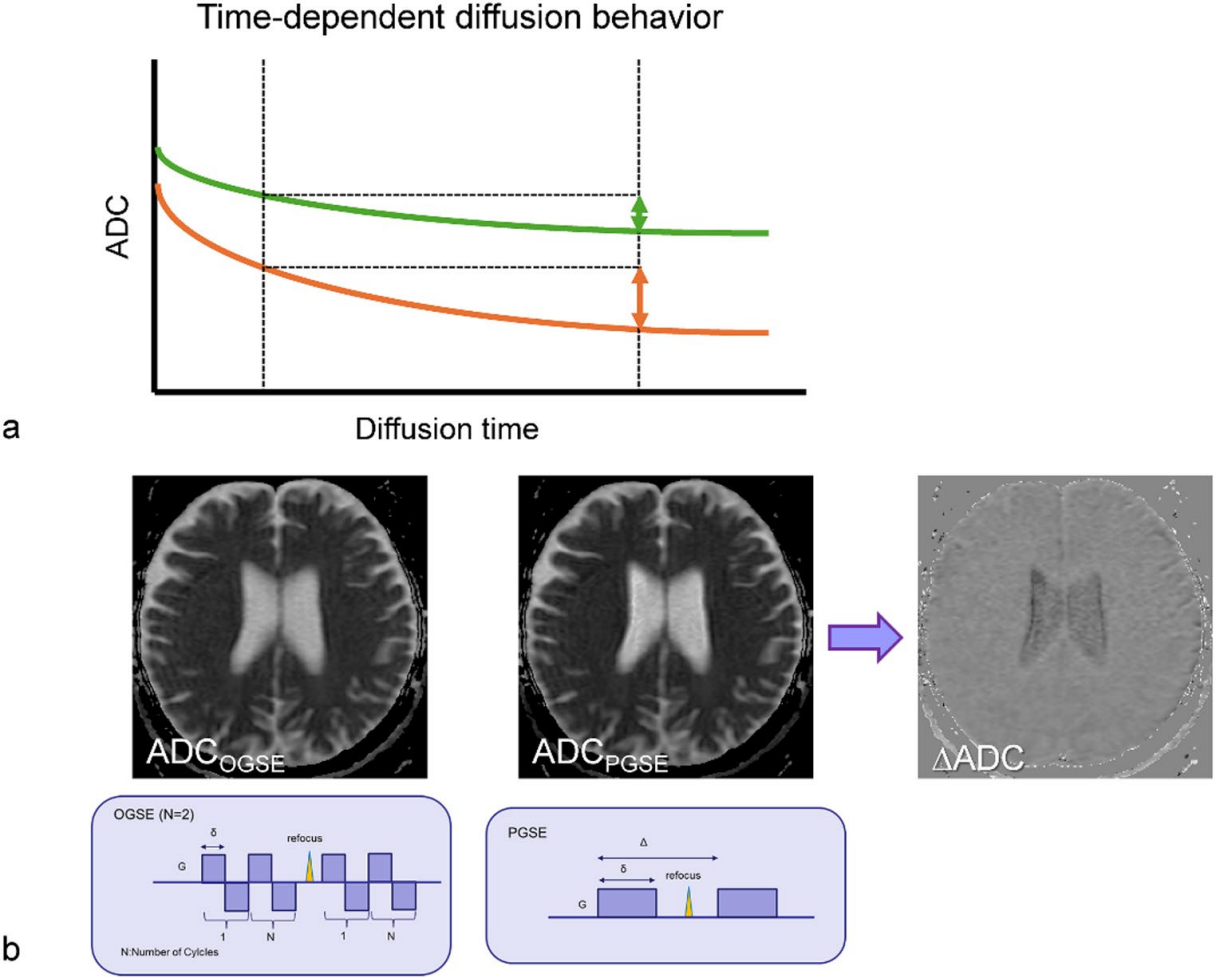
Concept of time-dependent diffusion and definition of the OGSE/PGSE-based difference metric (ΔOGSE–PGSE). **a** Schematic illustration of the concept of time-dependent diffusion, in which the apparent diffusion coefficient (ADC) varies as a function of diffusion time. The diagram conceptually demonstrates that ADC values change depending on diffusion time, indicating the presence of tissue properties that cannot be fully captured by diffusion MRI based on a single diffusion time. **b** Representative ADC maps obtained using oscillating gradient spin echo (OGSE) and pulsed gradient spin echo (PGSE), together with the definition of the difference metric. ΔOGSE–PGSE is defined as the difference between ADC derived from OGSE, which is sensitive to shorter diffusion times, and ADC derived from PGSE, which reflects relatively longer diffusion times (ΔADC = ADC_OGSE − ADC_PGSE). This metric is used as an index reflecting time-dependent diffusion characteristics. The waveform illustration represents a conceptual schematic rather than the exact hardware waveform implemented on the scanner

A key limitation of conventional DTI is its reliance on a single diffusion time, which restricts sensitivity to certain microstructural properties. In contrast, time-dependent diffusion MRI probes tissue microstructure across multiple spatial scales by varying diffusion time (Fig. 1a) [21]. Among these approaches, the difference between oscillating gradient spin echo (OGSE) and pulsed gradient spin echo (PGSE) diffusion sensitivities (ΔOGSE–PGSE) has been shown to capture microstructural features such as membrane permeability and cell size distribution that are not readily assessed using conventional diffusion models (Fig. 1b) [22–25].

OSA is a clinically relevant condition characterized by chronic physiological stress on the brain, including intermittent hypoxia and sleep fragmentation [26]. The severity of OSA is commonly quantified using polysomnography-derived indices. The apnea–hypopnea index (AHI), defined as the total number of apnea and hypopnea events per hour of sleep, is widely used as the standard clinical measure of OSA severity. The hypopnea index (HI) specifically reflects the frequency of hypopnea events per hour of sleep and may capture milder respiratory disturbances associated with partial airway obstruction. Although OSA does not typically cause overt focal lesions, it has been associated with widespread and subtle alterations in white matter microstructure, and has also been discussed in the context of glymphatic dysfunction [27]. Diffusion-based surrogate markers, including the ALPS index, have been reported to show severity-dependent changes in patients with OSA, making OSA a suitable in vivo model for examining the interplay between white matter microstructure, diffusion time–dependent metrics, and neurofluid-related imaging indices [28–31].

Based on this background, this study aimed to investigate associations between ΔOGSE–PGSE and sleep- and respiratory-related indices derived from PSG, as well as neurofluid-related imaging metrics, including relative choroid plexus volume (rCPV), relative white matter hyperintensity volume (rWMHV), and the ALPS index, as part of the second phase of the MOONLIGHT study. To address this aim, ΔOGSE–PGSE maps were spatially normalized to MNI space and analyzed using atlas-based region-of-interest methods to characterize whole-brain association patterns. In addition, exploratory ternary plot analyses stratified by the AHI and HI were performed to visualize relative signal composition and to provide contextual insight into the structural background relevant to interpretation of neurofluid-related imaging metrics.

## Materials and methods

### Participants

This prospective observational study was conducted at our institute between December 2020 and December 2024. Sixty-four participants with suspected sleep apnea were screened, and 50 participants who provided written informed consent and met eligibility criteria completed baseline MRI examinations and overnight polysomnography (PSG) [11].

Ten imaging or PSG sessions were excluded because of missing T1-weighted images (*n* = 4), incomplete OGSE or PGSE acquisitions (*n* = 5), or incomplete PSG data (*n* = 1). The final cohort consisted of 40 participants, contributing 80 MRI sessions (two sessions per participant: evening [d0] and following morning [d1]) along with their corresponding PSG datasets. All remaining sessions underwent visual quality control procedures. No additional sessions were excluded due to motion artifacts, inter-sequence misalignment, failed normalization, or other image quality concerns.

The mean age was 49.7 ± 13.3 years, and the cohort included 29 male and 11 female; all participants were diagnosed with obstructive sleep apnea. Body mass index (BMI) and psychiatric comorbidities (psychotic, mood, and obsessive–compulsive–related disorders) were obtained from electronic medical records. OSA severity was assessed using the AHI derived from PSG.

Each participant underwent MRI in the evening (d0, approximately 16:00), overnight PSG (> 8 h), and a second MRI the following morning (d1, approximately 09:00). To account for potential diurnal variation, imaging session (d0/d1) was included as a covariate in the statistical models. Age, sex, BMI, and psychiatric comorbidities were also included as covariates. Because vascular and metabolic factors can influence both obstructive sleep apnea severity and white matter microstructure, these variables were included to mitigate potential confounding effects. In addition, white matter hyperintensity (WMH) burden was considered as an imaging marker of small-vessel disease that may influence diffusion measures. Continuous variables were z-standardized, and binary variables were coded as 0 or 1.

The study was approved by the institutional review board (approval number: 2020 − 0352), registered in the UMIN Clinical Trials Registry (R000045230), and conducted in accordance with the Declaration of Helsinki. The study objectives and procedures were explained to all participants, and written informed consent was subsequently obtained from each participant.

### Polysomnography

Overnight PSG was performed using Embla N7000 (Natus, WI, USA) and PSG-1100 (Nihon Kohden Corporation, Tokyo, Japan). Recordings were obtained using a standard full-montage protocol in accordance with the recommendations of the American Academy of Sleep Medicine (AASM) [32]. The recorded signals included electroencephalography (EEG), electrooculography (EOG), chin electromyography (EMG), electrocardiography (ECG), nasal airflow measured by nasal cannula and thermistor, thoracic and abdominal respiratory effort, and transcutaneous arterial oxygen saturation (SpO₂).

Sleep stages and respiratory event scoring were performed according to the AASM Scoring Manual version 2.5. The following PSG-derived parameters were calculated for analysis: total sleep time (TST), sleep onset latency, rapid eye movement (REM) sleep latency, wake after sleep onset WASO, sleep efficiency, proportions of each sleep stage (N1, N2, N3, and REM), arousal index, HI, AHI, mean SpO₂, minimum SpO₂, and the 3% oxygen desaturation index (ODI3%).

### Imaging

Diffusion MRI was acquired using a 3-T clinical MRI system (Vantage Centurian, Canon Medical Systems, Tochigi, Japan). Time-dependent diffusion MRI used both oscillating gradient spin echo (OGSE) and pulsed gradient spin echo (PGSE) waveforms. To ensure signal comparability, imaging parameters were identical for both OGSE and PGSE acquisitions, except for the diffusion time. Imaging parameters were as follows: repetition time (TR), 6200 ms; echo time (TE), 120 ms; motion-probing gradients, monopolar; b values, 0 and 1000 s/mm². Slice thickness was 5 mm, field of view (FOV) was 220 mm, and matrix size was 128 × 160. The diffusion times were set to 6.94 ms for OGSE (corresponding to an oscillation frequency of 44 Hz) and 60 ms for PGSE.

OGSE and PGSE acquisitions were performed within the same imaging session using identical geometric parameters (field of view, matrix size, slice thickness, and slice orientation). ADC maps were reconstructed in the same native image space. Because both datasets shared identical geometry, additional spatial registration was not required prior to computing ΔOGSE–PGSE. Visual inspection confirmed the absence of gross inter-sequence misalignment. For the OGSE acquisition, diffusion encoding gradients were implemented using a sinusoidal oscillating waveform approximated by a trapezoidal gradient representation on the scanner hardware, with a gradient ramp time of 1500 µs. The oscillation parameter *N* = 2 represents two oscillation cycles per diffusion gradient lobe.

The effective diffusion time corresponding to the oscillation frequency was calculated using the Gaussian phase distribution approximation described by Ianuş et al. [33]. This formulation provides an analytical relationship between oscillation frequency and effective diffusion time for oscillating gradient spin-echo sequences. A summary of the same calculation framework has also been described in the Appendix of a previous technical validation study [34].

Diffusion preprocessing such as motion correction, eddy-current correction, or susceptibility distortion correction was not applied as separate steps, because ADC maps were reconstructed directly on the scanner from diffusion-weighted images acquired with identical acquisition geometry within the same session. The ΔOGSE–PGSE map was therefore computed from ADC maps generated within the same scanner reconstruction pipeline. All datasets were visually inspected to confirm the absence of motion artifacts and inter-sequence misalignment prior to analysis.

Apparent diffusion coefficients (ADCs) were calculated for both conditions, and a difference metric (ΔADC) was derived as ΔOGSE–PGSE = ADC_OGSE − ADC_PGSE, which was used for evaluation of tissue microstructural properties.

Separately, diffusion tensor imaging (DTI) was also acquired for the ALPS index calculation using a single-shot EPI sequence. Imaging parameters were as follows: TR, 5300 ms; TE, 73 ms; b values, 0 and 1000 s/mm²; number of diffusion gradient directions, 30. Slice thickness was 2 mm, FOV was 224 × 224 mm², and matrix size was 112 × 112.

As structural imaging, three-dimensional T1-weighted images were acquired using a magnetization-prepared rapid acquisition with gradient echo (MPRAGE) sequence. Imaging parameters were as follows: TR, 2300 ms; TE, 2.36 ms; inversion time (TI), 900 ms; flip angle, 9°; slice thickness, 1 mm; FOV, 256 × 256 mm²; and matrix size, 256 × 256.

### Analysis

#### Atlas-based ROI analysis

Atlas-based ROI values were extracted from ΔOGSE–PGSE maps spatially normalized to the MNI standard space (1-mm isotropic resolution). All analyses were performed using Python version 3.10. ROIs were defined using the Harvard–Oxford Cortical Atlas (maximum probability, threshold 25%), the Harvard–Oxford Subcortical Atlas (maximum probability), and the JHU ICBM-DTI-81 White Matter Labels, all at 1-mm resolution [35, 36]. A brain mask derived from the MNI152_T1 template was applied to exclude non-brain voxels.

Cortical ROIs were treated as bilateral regions according to atlas definitions, whereas subcortical and white matter ROIs were analyzed separately for the left and right hemispheres. For each participant and imaging session, ROI values were calculated as the arithmetic mean of ΔOGSE–PGSE voxel values within each atlas-defined region.

#### Assessment of session effects

To evaluate potential effects of imaging session timing, analyses were conducted using data acquired before CPAP treatment. Participants with complete MRI data for both sessions (d0 and d1) were included. For each ROI, linear mixed-effects models were fitted with the ROI value as the dependent variable, imaging session (d0 or d1) as a fixed effect, and participant as a random effect. Paired t-tests were additionally performed in the same subset as a complementary analysis. Multiple comparisons were corrected using the Holm method and the Benjamini–Hochberg false discovery rate (FDR), and effect sizes were calculated using Cohen’s d.

#### Cross-sectional analysis

The primary hypothesis of this study was that obstructive sleep apnea severity is associated with time-dependent diffusion changes measured using ΔOGSE–PGSE. The apnea–hypopnea index (AHI) was treated as the primary PSG predictor because it represents the standard clinical measure of OSA severity. Atlas-based ROI analyses focusing on white matter and subcortical regions were regarded as the primary analysis set, whereas analyses involving additional PSG metrics (e.g., hypopnea index), cortical ROIs, and neurofluid-related imaging metrics were considered exploratory.

Cross-sectional analyses were performed using data acquired from 40 participants (80 imaging sessions) to examine associations between the diffusion metric difference (ΔOGSE–PGSE) in each ROI and PSG-derived indices as well as neurofluid-related imaging metrics. ROIs were selected with an emphasis on subcortical structures and major white matter pathways previously implicated in glymphatic function and white matter integrity.

Associations were assessed using linear mixed-effects models, with ΔROI values as the dependent variable and each PSG-derived or neurofluid-related metric entered separately as the independent variable. Age, sex, BMI, and psychiatric diagnosis were included as covariates, and participant ID was modeled as a random effect. As no significant differences were observed between d0 and d1 sessions, data from both sessions were pooled. Multiple comparisons across ROIs were corrected for each index using the Holm method and the FDR.

To assess the potential influence of small-vessel disease burden, additional sensitivity analyses were performed by including white matter hyperintensity volume (rWMHV) as a covariate in the regression models.

**Fig. 2:**
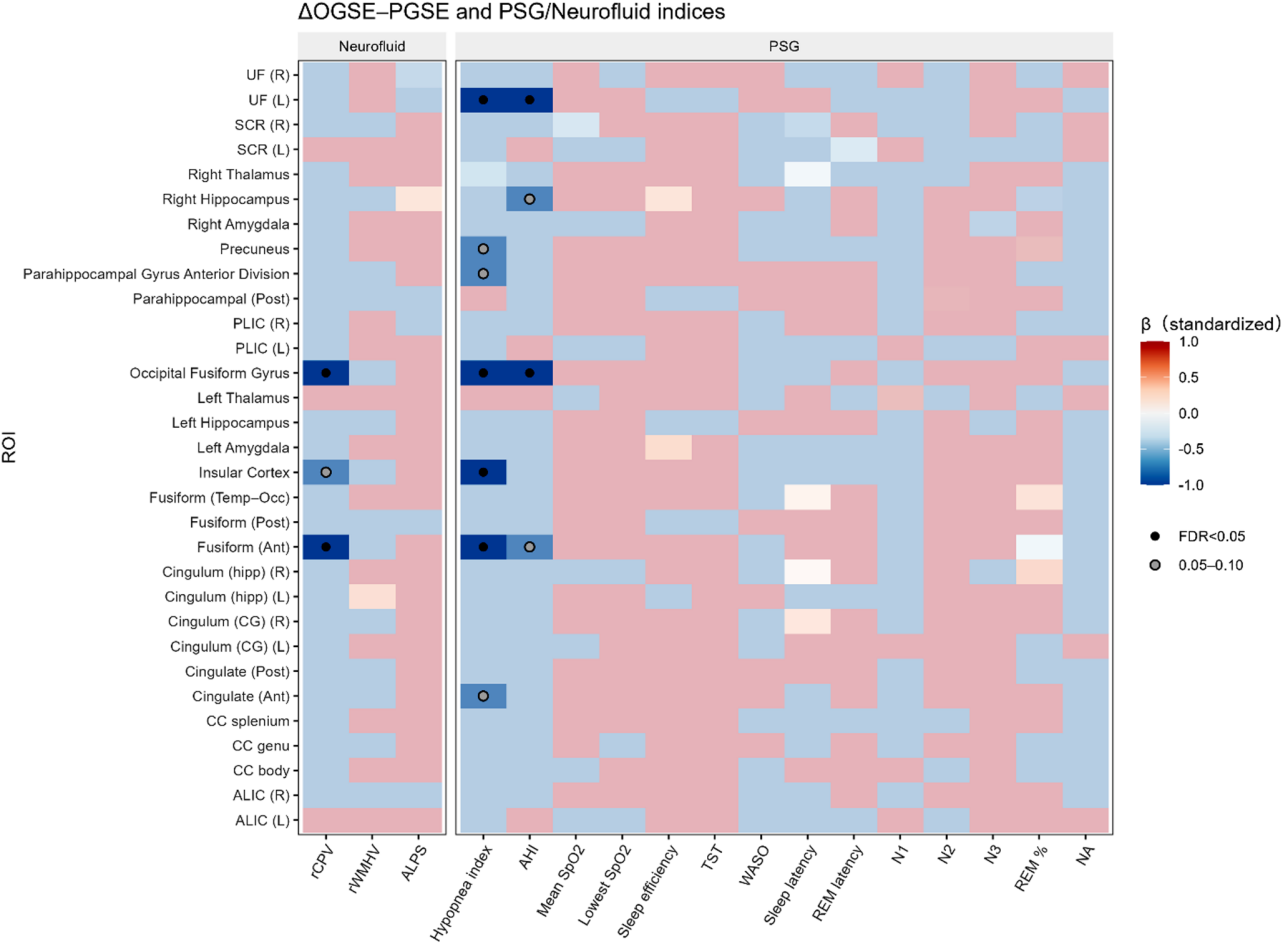
Associations of ΔOGSE–PGSE with polysomnography (PSG)–derived indices and neurofluid-related imaging metrics across regions of interest (ROIs). Associations between ΔOGSE–PGSE and imaging or polysomnography (PSG)–derived indices within predefined ROIs are shown as standardized regression coefficients (β). The left panel shows associations with neurofluid-related imaging metrics (relative choroid plexus volume [rCPV], relative white matter hyperintensity volume [rWMHV], and the ALPS index), while the right panel shows associations with PSG-derived respiratory and sleep-related indices, including the HI, AHI, SpO₂-related indices, sleep efficiency, total sleep time, arousal index, sleep latency, REM latency, and sleep stage proportions. Color indicates the direction and magnitude of standardized β values (red, positive; blue, negative), with lighter colors representing smaller absolute effect sizes. Statistically significant associations after false discovery rate (FDR) correction (qFDR < 0.05) are marked with black circles (●), and trend-level associations (0.05 ≤ qFDR < 0.10) with gray circles (○). ROI labels follow the Harvard–Oxford cortical and subcortical atlases and the JHU white matter atlas, using standard abbreviations. Corpus callosum (CC genu, CC body, CC splenium); cingulum (cingulate gyrus portion: CG; hippocampal portion: hipp); uncinate fasciculus (UF); internal capsule (anterior limb: ALIC; posterior limb: PLIC); superior corona radiata (SCR); cingulate gyrus (anterior/posterior); fusiform gyrus (anterior/posterior, temporo-occipital); posterior division of the parahippocampal gyrus (Parahippocampal post); and precuneus cortex (Precuneus)

**Fig. 3:**
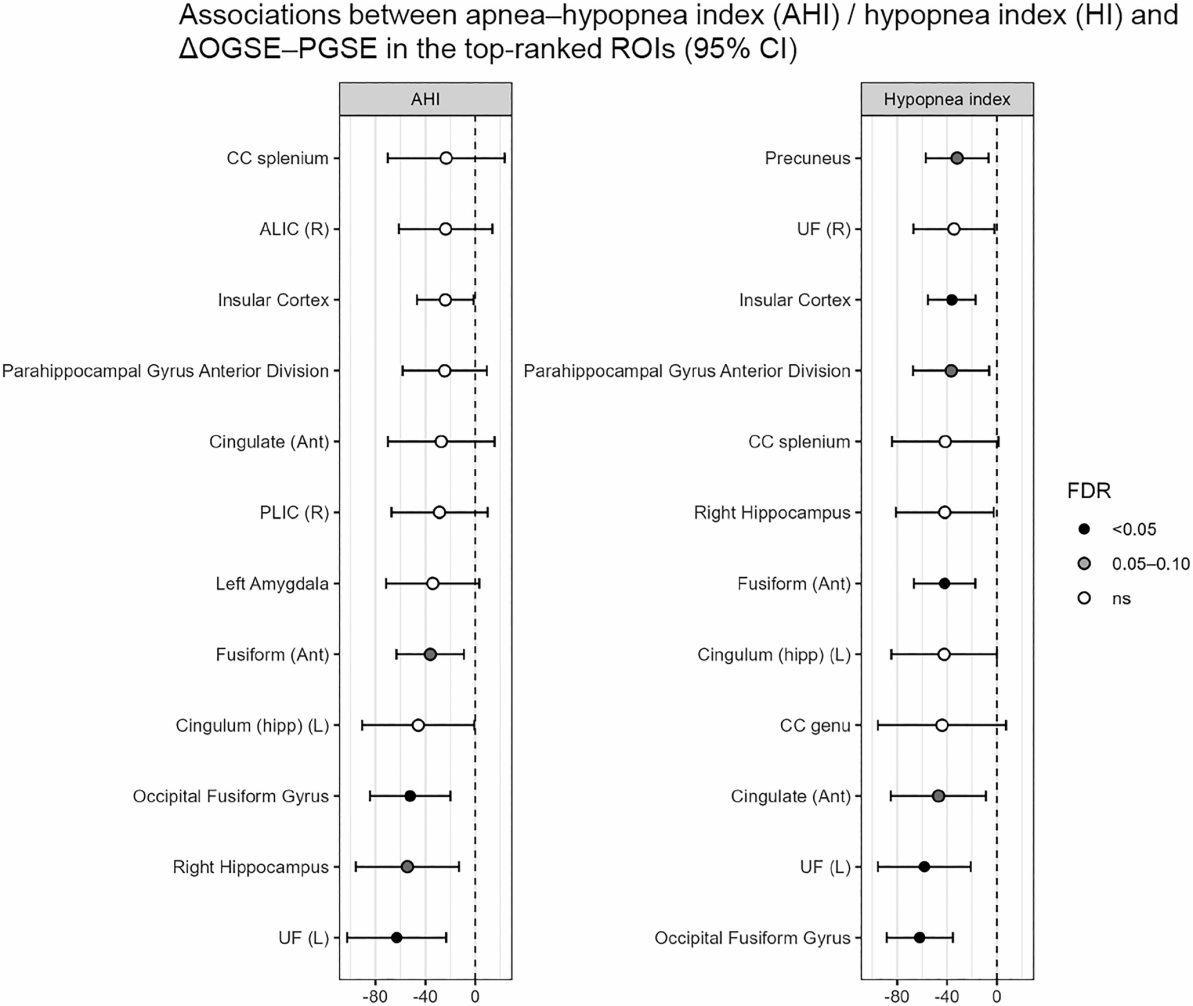
Forest plots showing associations between the hypopnea index (HI), apnea–hypopnea index (AHI), and ΔOGSE–PGSE. Forest plots show associations between the hypopnea index (HI) and the apnea–hypopnea index (AHI) and the time-dependent diffusion MRI metric ΔOGSE–PGSE, based on atlas-based ROI analyses. Points indicate standardized regression coefficients (β) estimated using linear mixed-effects models, with horizontal bars representing 95% confidence intervals. The displayed ROIs correspond to the 12 regions with the largest absolute effect sizes (|β|) for each index. ROIs with statistically significant associations after false discovery rate (FDR) correction (qFDR < 0.05) are shown as black circles, trend-level associations (0.05 ≤ qFDR < 0.10) as gray circles, and non-significant associations as open circles. For the HI, associations were observed in regions including the insular cortex, anterior fusiform gyrus, and precuneus, whereas for AHI, associated regions included the splenium of the corpus callosum, anterior limb of the internal capsule, and amygdala, indicating partially distinct association patterns across severity metrics

**Fig. 4:**
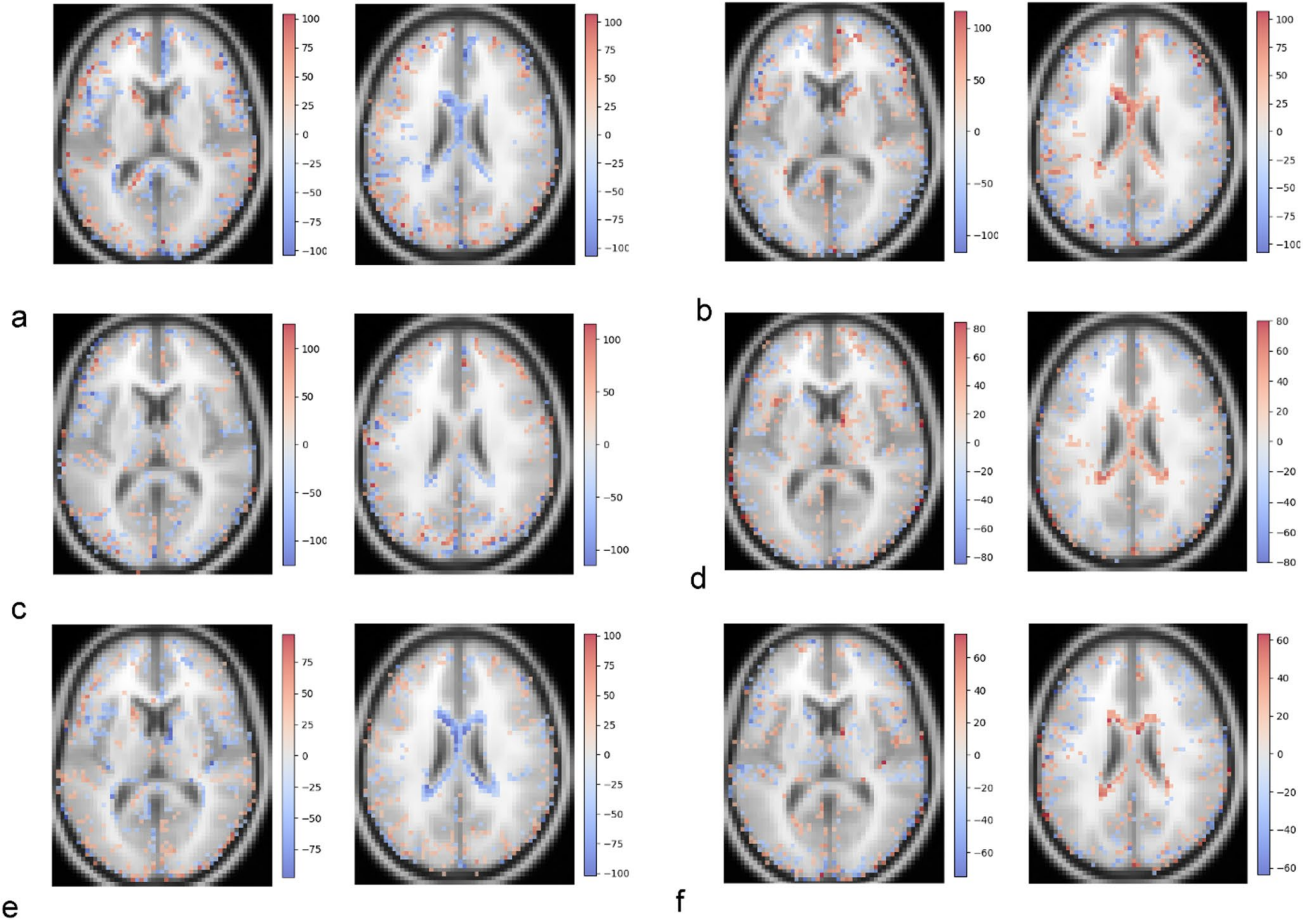
Voxel-based standardized β maps of associations between ΔOGSE–PGSE and representative PSG and neurofluid indices. Voxel-wise spatial distributions of standardized regression coefficients (β) for associations between ΔOGSE–PGSE and selected indices, estimated using linear mixed-effects models, are shown. Representative respiratory indices include **a** the HI (negative association) and **b** minimum SpO₂ (positive association); sleep-related indices include **c** sleep latency (negative association) and **d** sleep efficiency (positive association); and neurofluid-related indices include **e** relative choroid plexus volume (rCPV; negative association) and **f** the ALPS index (positive association). Maps are displayed within the analysis mask, showing voxels in the upper 25% of absolute β values (≥ 75th percentile), with clusters smaller than 30 voxels excluded. Warm colors indicate positive associations and cool colors indicate negative associations. Standard T1-weighted images are shown in the background, with outlines of the Harvard–Oxford cortical and subcortical atlases and the JHU white matter atlas overlaid. No voxel clusters survived correction for multiple comparisons; therefore, these maps are presented as exploratory visualizations to complement the atlas-based ROI analysis

Results were summarized using heatmaps showing standardized regression coefficients and FDR-adjusted significance levels (Fig. 2). For selected representative indices, ROIs with larger effect sizes were further evaluated using forest plots with 95% confidence intervals (Fig. 3). In addition, voxel-based analyses using the same statistical framework were performed as a supplementary visualization of spatial association patterns (Fig. 4).

#### Sensitivity analyses

To evaluate the potential impact of partial-volume effects associated with relatively thick diffusion slices and spatial normalization, a sensitivity analysis using an ROI erosion strategy was performed. All atlas-defined ROIs were subjected to a one-voxel three-dimensional morphological erosion prior to extraction of ΔOGSE–PGSE values. This procedure removes boundary voxels that are most susceptible to partial-volume contamination from cerebrospinal fluid or adjacent tissue compartments. ROI mean ΔOGSE–PGSE values were then recalculated using the eroded ROIs, and the statistical analyses were repeated using the same models and covariates as in the primary analysis.

To account for within-subject dependence arising from the paired imaging sessions (d0 and d1), an additional sensitivity analysis was performed using cluster-robust standard errors with subject ID specified as the clustering variable. The results of these sensitivity analyses are summarized in Supplementary Tables.

#### Supplementary ternary plot analysis

To visualize signal changes underlying ΔOGSE–PGSE as relative compositions of multiple MRI-derived metrics, a ternary plot analysis was performed as an exploratory visualization approach [37]. This analysis was not intended for group discrimination or statistical testing, but to provide an intuitive overview of signal composition patterns within ROIs.

Three MRI-derived metrics were included: OGSE-derived apparent diffusion coefficient (ADC), reflecting short diffusion-time sensitivity; the PGSE/OGSE ADC ratio, representing relative diffusion-time–dependent structural sensitivity; and the b0 signal, included as a surrogate of water content. For each ROI, these metrics were calculated voxelwise and normalized so that the sum of the three components equaled 1, enabling visualization within a ternary coordinate system.

ROIs were selected based on the atlas-based cross-sectional analysis, focusing on the three regions showing the strongest associations with ΔOGSE–PGSE for each index. Participants were stratified according to PSG-derived indices: for AHI, a higher (AHI ≥ 20) and lower (AHI < 20) group; and for HI, a higher (HI ≥ 15) and lower (HI < 15) group. These stratifications were applied independently for visualization.

All analyses were performed on diffusion images normalized to MNI space, with spatial resolution unified to 2-mm isotropic voxels. ADC_OGSE, ADC_PGSE, and b0 images were processed on the same spatial grid.

## Results

### Session effects

In the 40 participants, no significant differences were observed between the d0 and d1 imaging sessions for any ROI. Linear mixed-effects models revealed no significant session effects across all ROIs, and the results of the paired t-tests were consistent with these findings. Effect sizes, as quantified by Cohen’s d, were uniformly small. Based on these results, no systematic differences between the d0 and d1 sessions were identified. Therefore, data from both sessions were pooled for subsequent analyses.

### Cross-sectional analysis

#### Atlas-based associations with PSG and neurofluid indices

In the primary analysis focusing on white matter and subcortical regions with AHI as the main predictor, significant associations with ΔOGSE–PGSE were identified across multiple ROIs (Fig. 2).

In exploratory analyses, the HI demonstrated significant negative associations with ΔOGSE–PGSE in the insular cortex, anterior temporal fusiform cortex, occipital fusiform gyrus, and the left uncinate fasciculus (all qFDR < 0.05). Among these regions, the association observed in the occipital fusiform gyrus was the strongest. AHI also showed significant negative associations with ΔOGSE–PGSE in the occipital fusiform gyrus and the left uncinate fasciculus (Fig. 3, qFDR < 0.05).

In exploratory analyses, negative associations between the HI and ΔOGSE–PGSE were also observed at a trend level in the anterior cingulate cortex, precuneus, and anterior parahippocampal gyrus (qFDR < 0.10).

Among the neurofluid-related imaging metrics, rCPV showed significant negative associations with ΔOGSE–PGSE in the anterior temporal fusiform cortex and the occipital fusiform gyrus, which remained significant after FDR correction (Figs. 2 and 3, qFDR < 0.01). A similar association was observed in the insular cortex, although it did not reach the corrected significance threshold of qFDR < 0.05. No ROIs showed significant associations with ΔOGSE–PGSE for rWMHV or the ALPS index after FDR correction. Sensitivity analyses using ROI erosion and cluster-robust standard errors yielded consistent association patterns, indicating that the main findings were not driven by boundary-related partial-volume effects or within-subject dependence (Supplementary Tables). In additional sensitivity analyses including rWMHV as a covariate, the overall pattern of associations remained unchanged, indicating that the main findings were not driven by white matter hyperintensity burden.

### Voxel-based analysis with PSG and neurofluid indices

In the voxel-based analysis, the same covariate-adjusted linear mixed-effects models used in the ROI-based analysis were applied at each voxel to generate maps of standardized regression coefficients (β). Trend-level spatial distribution patterns associated with sleep-related indices, respiratory indices, and neurofluid-related metrics were observed, primarily involving the basal ganglia and the body of the corpus callosum.

For respiratory indices, the HI, AHI, and arousal index showed negative associations with ΔOGSE–PGSE, whereas minimum SpO₂ exhibited positive associations. Regarding sleep-related indices, sleep latency and REM latency were negatively associated with ΔOGSE–PGSE, while total sleep time and sleep efficiency showed positive associations. Among the neurofluid-related metrics, rCPV and rWMHV demonstrated negative association patterns extending from the basal ganglia toward the corpus callosum, whereas the ALPS index exhibited a predominantly positive association pattern.

No statistically significant voxel clusters survived multiple comparison correction. For this reason, the primary conclusions of this study were based on the atlas-based ROI analysis. The voxel-based results served to complement the ROI-level findings by revealing consistent trend-level spatial distributions in the basal ganglia and corpus callosum, in addition to the insular and temporal regions identified in the ROI analysis. As representative examples, standardized β maps are presented in Fig. 4 for the HI (negative association) and minimum SpO₂ (positive association) among respiratory indices, for sleep latency (negative association) and sleep efficiency (positive association) among sleep-related indices, and for rCPV (negative association) and the ALPS index among neurofluid-related metrics.

### Supplementary ternary plot analysis

**Fig. 5:**
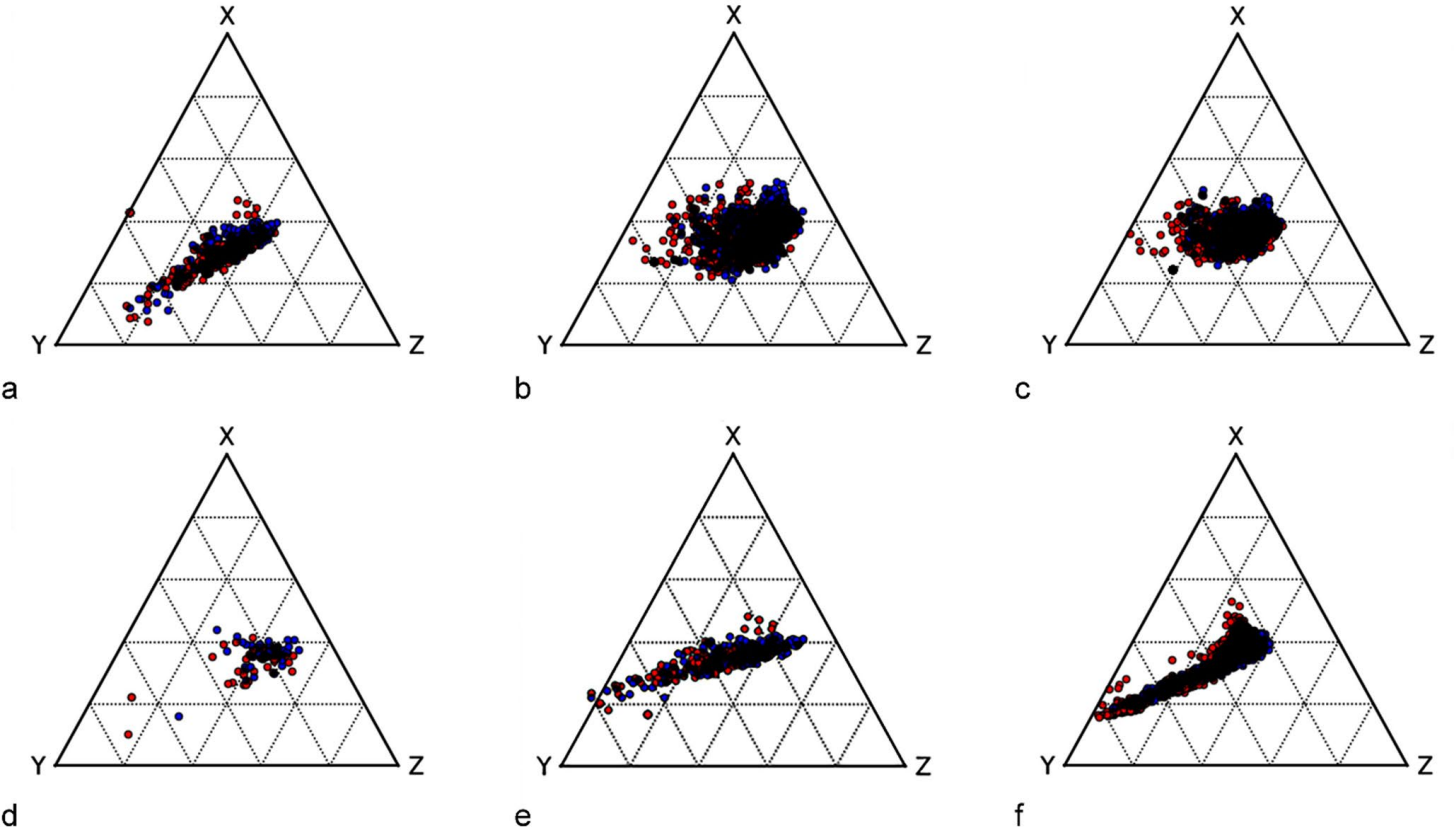
Exploratory ternary plots of diffusion-related signal composition stratified by apnea–hypopnea index (AHI) and hypopnea index (HI). Ternary plots illustrate the relative composition of the PGSE/OGSE ratio, OGSE-derived ADC, and b0 signal for all voxels within selected ROIs. Stratification by AHI is shown for **a** the left uncinate fasciculus, **b** the right hippocampus, and **c** the temporo-occipital fusiform gyrus, and stratification by HI is shown for **d** the anterior cingulate cortex, **e** the right hippocampus, and **f** the temporo-occipital fusiform gyrus. Blue and red denote lower and higher index groups, respectively. Across all ROIs, voxel distributions from the two groups largely overlap and are aligned along a common compositional axis, indicating continuous variation in relative signal composition rather than distinct clustering. No isolated dominance of a single signal component was observed. This ternary plot analysis was not intended for quantitative comparison or statistical testing and is presented solely as an exploratory visualization to aid interpretation of the atlas-based ROI results

To further characterize the components underlying ΔOGSE–PGSE, a ternary plot analysis was performed as an exploratory visualization of relative signal composition using three MRI-derived metrics: the ADC_PGSE/ADC_OGSE ratio, OGSE-derived ADC, and the b0 signal (Fig. 5).

When stratified by the AHI, voxel distributions from the lower and higher AHI groups did not form clearly separated clusters on the ternary plot, but instead occupied a shared elongated distribution band. This band followed a coordinated direction characterized by increasing PGSE/OGSE ratio, decreasing OGSE ADC, and a relative increase in the b0 signal. Compared with the lower AHI group, the higher AHI group exhibited a subtle shift in the centroid of the distribution along this coordinated direction. No divergence attributable to a preferential increase in the b0 signal alone was observed. These distributional features were consistently observed across the left uncinate fasciculus, right hippocampus, and the occipitotemporal fusiform gyrus ROIs.

In stratification based on the HI, voxel distributions from the higher and lower HI groups also largely overlapped and aligned along the same coordinated direction. However, the higher HI group demonstrated a broader distribution band, indicating increased dispersion along the shared direction. This relative increase in distributional width was more pronounced in cortical-associated ROIs.

## Discussion

The present study represents a direct extension of the first phase of the MOONLIGHT study, which addressed the structural dependency underlying interpretation of the DTI-ALPS method. In that phase, significant associations between DTI-derived metrics and the ALPS index suggested that ALPS-index may be influenced by white matter microstructural properties [11]. Building on this framework, the current study focused on the time-dependent diffusion metric ΔOGSE–PGSE, which is sensitive to microstructural features not fully captured by conventional DTI [21, 38–40], and examined its associations in participants with OSA. In the primary analysis focusing on white matter and subcortical regions, ΔOGSE–PGSE demonstrated significant associations with the apnea–hypopnea index (AHI). In exploratory analyses, additional associations were observed for the hypopnea index (HI) and several cortical regions, including the insular and temporal cortices. In contrast, associations with neurofluid-related imaging metrics were limited: significant relationships were observed only for rCPV in selected ROIs, whereas no significant associations were detected for the ALPS index or rWMHV. Supplementary voxel-based and ternary plot analyses showed trends consistent with the primary ROI-level findings. In combination, these results indicate that ΔOGSE–PGSE is broadly associated with white matter microstructural alterations related to OSA and, while it does not directly reflect neurofluid function, may capture structural background factors that may influence the interpretation of neurofluid-related imaging metrics, including the ALPS index. The primary interpretation of this study is therefore based on the associations between ΔOGSE–PGSE and AHI in white matter and subcortical regions. Findings involving HI and cortical regions should be considered exploratory and interpreted with appropriate caution. The relationship between these findings and neurofluid-related imaging metrics should therefore be interpreted cautiously and remains to be further validated.

The ALPS method was originally proposed as a non-invasive diffusion MRI–based approach to evaluate glymphatic function by capturing anisotropic water diffusion along perivascular spaces and has since been applied to a wide range of conditions, including aging, dementia, and sleep-related disorders [2–6, 9–13]. As a diffusion MRI–derived metric, however, the ALPS index may reflect not only perivascular water dynamics but also the influence of surrounding white matter microstructure [15, 18]. Because the ALPS index is calculated from the diffusion tensor model, DTI-sensitive structural factors such as fiber orientation, tissue organization, and microstructural integrity may contribute to its values [14, 16]. Recent reviews have further clarified this issue, emphasizing that the ALPS index should be interpreted as a surrogate measure incorporating structural background effects rather than as a pure marker of perivascular water dynamics [19, 20]. Accurate interpretation of ALPS index changes thus requires a complementary assessment of the surrounding white matter microstructure alongside diffusion properties along perivascular spaces. To this end, the time-dependent diffusion metric ΔOGSE–PGSE employed in the present study, which is sensitive to microstructural features not fully captured by conventional DTI, may provide a useful framework for examining the structural dependency underlying the DTI-ALPS method [21, 38–41].

In the present study, ΔOGSE–PGSE showed significant associations with the HI and the AHI across multiple regions, including the insular cortex, temporal lobe–related cortices, and the uncinate fasciculus, indicating that microstructural alterations associated with OSA can be detected at the level of white matter. As a time-dependent diffusion MRI metric, ΔOGSE–PGSE reflects differences in diffusivity across distinct diffusion times and is therefore sensitive to microstructural features that are not fully reflected by conventional DTI. Whereas standard DTI metrics primarily reflect diffusion anisotropy and mean diffusivity, their sensitivity to restricted diffusion and membrane-related effects that emerge in a diffusion time–dependent manner is limited. Importantly, this diffusion-time–dependent contrast is not directly captured by conventional diffusion tensor imaging acquired at a single diffusion time. Metrics such as FA, MD, RD, and AD primarily characterize diffusion anisotropy and overall diffusivity but do not directly probe how diffusivity changes across diffusion time scales. In this context, ΔOGSE–PGSE provides complementary information that reflects the time dependence of water diffusion and may therefore capture microstructural features that are not fully represented by conventional tensor-derived measures. In contrast, ΔOGSE–PGSE—defined as the difference between OGSE measurements sensitive to shorter diffusion times and PGSE measurements reflecting longer diffusion times—can be positioned as a metric that extracts complementary microstructural information through diffusion time dependence. From this perspective, ΔOGSE–PGSE may complement the structural dependency inherent in conventional diffusion MRI–based metrics, including the DTI-ALPS method. From a pathophysiological perspective, such microstructural alterations may reflect chronic stress responses induced by intermittent hypoxia and sleep fragmentation in obstructive sleep apnea, potentially influencing myelin integrity and axonal membrane–related microstructural environments without causing overt tissue loss [42]. These changes may preferentially influence diffusion behavior at shorter diffusion times, rendering ΔOGSE–PGSE sensitive to subclinical microstructural alterations associated with disease severity, although this interpretation remains hypothetical.

Analysis of distributional patterns revealed that the associations between respiratory event severity and ΔOGSE–PGSE differed depending on the index used: AHI was primarily associated with shifts in distributional position, whereas HI was characterized by an expansion of distributional width, suggesting heterogeneity in how these indices relate to white matter microstructural alterations. OSA chronically imposes physiological stress on the brain through mechanisms such as intermittent hypoxia and sleep fragmentation [43, 44]. Although these stressors do not typically produce overt focal lesions, they are thought to exert cumulative effects on white matter microstructure over time, resulting in widespread but subtle alterations [45–47]. This diffusion time dependence likely renders ΔOGSE–PGSE particularly sensitive to such subclinical structural changes. Our findings suggest that OSA imposes a chronic “structural load” on white matter; ΔOGSE–PGSE thus offers a unique tool to detect and characterize these effects in vivo. Notably, the association between higher OSA severity and reduced ΔOGSE–PGSE points to a loss of diffusion time-dependence, potentially reflecting altered microstructural complexity. Increased membrane permeability weakens the effect of restriction, resulting in reduced time-dependence [21]. Thus, the decrease in ΔOGSE–PGSE may reflect, at least in part, such permeability changes or a loss of microstructural complexity in severe OSA, although this interpretation remains speculative.

Regions showing significant associations with OSA severity were consistently observed in the insular cortex, temporally related cortical areas including the anterior temporal fusiform cortex and occipitotemporal fusiform gyrus, and the uncinate fasciculus. These regions are anatomically proximate and interconnected by association fibers supporting corticocortical and corticolimbic connectivity, with the uncinate fasciculus serving as a major long-range tract linking the anterior temporal lobe to the orbitofrontal region and connecting medial and lateral temporal cortices with the frontal lobe [48]. The concurrent involvement of cortical regions and the uncinate fasciculus suggests that the observed effects are not confined to focal cortical alterations but instead reflect structurally coherent changes spanning long-range white matter pathways. Because the present study did not assess functional connectivity or neural activity, these findings do not permit direct inferences regarding specific functional networks or underlying pathophysiological mechanisms. Rather, the structural implications are limited to the observation that microstructural alterations associated with OSA were detected across interconnected cortical–white matter pathways rather than in isolated regions. Interestingly, associations with ΔOGSE–PGSE were more consistently observed for the hypopnea index (HI) than for the apnea–hypopnea index (AHI). One possible explanation is that hypopnea events represent partial airway obstruction associated with sustained reductions in airflow and oxygenation, which may exert more prolonged physiological stress on brain tissue compared with brief apnea events. Alternatively, HI may reflect milder but more frequent respiratory disturbances, potentially capturing cumulative physiological burden that is relevant to subtle white matter microstructural alterations. However, the present findings should be interpreted cautiously, and further studies are required to clarify the physiological mechanisms underlying these associations.

In this study, ternary plot analysis was employed as a supplementary, exploratory approach to visualize ΔOGSE–PGSE–related signal changes in terms of the relative composition of the PGSE/OGSE ratio, OGSE-derived ADC, and the b0 signal, with the aim of facilitating intuitive understanding of signal composition patterns rather than group discrimination or classification. For both AHI– and HI–based stratification, voxel distributions did not form discrete clusters but instead exhibited continuous distributions aligned along a shared coordinated direction. This pattern suggests that ΔOGSE–PGSE–related changes are expressed as shifts in the relative balance among multiple diffusion- and signal-related metrics rather than through the emergence of a single dominant component. The PGSE/OGSE ratio, OGSE ADC, and b0 signal varied in a coordinated manner, and no isolated dominance or independent variability of the b0 signal was observed. The absence of independent b0-driven shifts argues against isolated changes in perivascular or interstitial water content. Notably, these ternary plot results represent qualitative observations of distributional patterns. They should therefore be viewed as complementary to the atlas-based ROI analysis, which remains the primary basis for the study’s conclusions.

Several limitations of this study should be acknowledged. The analyses were primarily cross-sectional, and therefore the observed associations between ΔOGSE–PGSE and indices of sleep and respiratory events do not allow causal inference. In addition, associations with neurofluid-related imaging metrics were limited, and the physiological mechanisms underlying ΔOGSE–PGSE–related signal changes remain to be clarified. Future studies employing larger cohorts and longitudinal designs will be necessary to further elucidate the clinical significance of time-dependent diffusion metrics and to better define their role in evaluating microstructural alterations associated with OSA. Although the diffusion acquisition used relatively thick slices, sensitivity analyses using ROI erosion demonstrated that the main association patterns were robust to potential partial-volume effects. Because OSA populations frequently present with vascular and metabolic comorbidities, residual confounding related to vascular risk factors cannot be completely excluded despite adjustment for demographic and metabolic covariates. Because the primary aim of the present study was to investigate time-dependent diffusion behavior, direct comparisons with conventional DTI-derived metrics (e.g., FA, MD, RD, or AD) were not included. Future studies directly comparing time-dependent diffusion metrics with standard tensor-derived measures may help further clarify their incremental value in characterizing microstructural alterations associated with OSA. Because the atlas ROIs were originally defined at a higher spatial resolution than the diffusion data, partial-volume effects at ROI boundaries cannot be completely excluded. However, sensitivity analyses using ROI erosion yielded consistent association patterns, suggesting that the main findings were not primarily driven by boundary-related partial-volume effects.

In conclusion, this study demonstrates that ΔOGSE–PGSE, a time-dependent diffusion MRI metric, reflects white matter microstructural alterations associated with OSA. These alterations encompass microstructural features that may not be fully captured by conventional diffusion MRI metrics, indicating that ΔOGSE–PGSE is sensitive to distinct features of white matter organization. Although ΔOGSE–PGSE does not directly assess glymphatic function or intracerebral fluid dynamics, it elucidates the shared microstructural substrates that influence diffusion-based neurofluid imaging indices, including the DTI-ALPS method. From a clinical perspective, time-dependent diffusion metrics such as ΔOGSE–PGSE offers a practical means to characterize white matter vulnerability and refine the interpretation of diffusion-based neurofluid imaging indices.

## Electronic Supplementary Material

Below is the link to the electronic supplementary material.

Supplementary Material 1

## Data Availability

The data supporting the findings of this study are available from the corresponding author upon reasonable request.
