# Supplementary material for: White matter microstructural alterations in obstructive sleep apnea assessed by time-dependent diffusion MRI
Source: Jpn J Radiol. 2026 Apr 16;44(8):1368–81. doi: 10.1007/s11604-026-01991-x (PMC13400672; doi:10.1007/s11604-026-01991-x)
Supplement: Supplementary file 1 — Supplementary Material 1 [file 11604_2026_1991_MOESM1_ESM.docx]

**Supplementary Tables**

**Supplementary Table S1（ROI erosion）**

**Sensitivity analysis using ROI erosion to mitigate partial-volume effects**

| ROI | PSG index | beta | p | qFDR |
| --- | --- | --- | --- | --- |
| Occipital Fusiform Gyrus | 3% Oxygen Desaturation Index | -53.306 | 0.0022 | 0.0341 |
| Uncinate fasciculus L | 3% Oxygen Desaturation Index | -69.092 | 0.0015 | 0.0341 |
| Right Hippocampus | 3% Oxygen Desaturation Index | -60.956 | 0.0049 | 0.0503 |
| Occipital Fusiform Gyrus | Apnea-Hypopnea Index (AHI) | -54.383 | 0.0012 | 0.0202 |
| Uncinate fasciculus L | Apnea-Hypopnea Index (AHI) | -67.743 | 0.0013 | 0.0202 |
| Right Hippocampus | Apnea-Hypopnea Index (AHI) | -59.563 | 0.0045 | 0.0468 |
| Temporal Fusiform Cortex anterior division | Apnea-Hypopnea Index (AHI) | -36.947 | 0.0078 | 0.0608 |
| Occipital Fusiform Gyrus | Hypopnea Index | -64.309 | 0.0 | 0.0003 |
| Insular Cortex | Hypopnea Index | -38.254 | 0.0001 | 0.002 |
| Temporal Fusiform Cortex anterior division | Hypopnea Index | -42.866 | 0.0008 | 0.0085 |
| Uncinate fasciculus L | Hypopnea Index | -60.413 | 0.0022 | 0.0171 |
| Cingulate Gyrus anterior division | Hypopnea Index | -48.903 | 0.008 | 0.0416 |
| Precuneous Cortex | Hypopnea Index | -33.141 | 0.0078 | 0.0416 |
| Parahippocampal Gyrus anterior division | Hypopnea Index | -38.497 | 0.0113 | 0.0501 |
| Right Hippocampus | Hypopnea Index | -45.427 | 0.0216 | 0.0836 |

Results of the sensitivity analysis after applying a one-voxel erosion to atlas-defined regions of interest (ROIs) to reduce potential partial-volume contamination.

β indicates the standardized regression coefficient derived from the mixed-effects model.

p values are two-sided and q values represent false discovery rate (FDR)–adjusted p values using the Benjamini–Hochberg procedure.

ROI: region of interest

PSG: polysomnography

AHI: apnea–hypopnea index

ODI: oxygen desaturation index

**Supplementary Table S2（cluster robust SE）**

**Sensitivity analysis using cluster-robust standard errors**

| ROI | PSG index | beta | SE (model) | SE (CR2) | p (model) | p (CR2) | qFDR (CR2) |
| --- | --- | --- | --- | --- | --- | --- | --- |
| Anterior limb of internal capsule L | 3% Oxygen Desaturation Index | 25.119 | 27.802 | 33.61 | 0.3716 | 0.4712 | 0.709 |
| Anterior limb of internal capsule R | 3% Oxygen Desaturation Index | -19.598 | 17.962 | 18.264 | 0.2818 | 0.3074 | 0.6207 |
| Body of corpus callosum | 3% Oxygen Desaturation Index | -9.191 | 18.784 | 16.222 | 0.626 | 0.583 | 0.7551 |
| Cingulate Gyrus anterior division | 3% Oxygen Desaturation Index | -29.335 | 20.326 | 24.511 | 0.1564 | 0.2575 | 0.6139 |
| Cingulate Gyrus posterior division | 3% Oxygen Desaturation Index | -18.29 | 18.566 | 13.849 | 0.3302 | 0.2147 | 0.5547 |
| Cingulum cingulate gyrus L | 3% Oxygen Desaturation Index | -9.389 | 21.586 | 18.983 | 0.6658 | 0.6311 | 0.7551 |
| Cingulum cingulate gyrus R | 3% Oxygen Desaturation Index | -19.593 | 15.941 | 11.465 | 0.2226 | 0.117 | 0.4982 |
| Cingulum hippocampus L | 3% Oxygen Desaturation Index | -38.422 | 24.517 | 24.874 | 0.1248 | 0.1519 | 0.4982 |
| Cingulum hippocampus R | 3% Oxygen Desaturation Index | -8.027 | 24.079 | 19.054 | 0.7405 | 0.682 | 0.7551 |
| Genu of corpus callosum | 3% Oxygen Desaturation Index | -10.85 | 27.176 | 25.612 | 0.6918 | 0.6803 | 0.7551 |
| Insular Cortex | 3% Oxygen Desaturation Index | -25.216 | 11.101 | 15.887 | 0.0283 | 0.1419 | 0.4982 |
| Left Amygdala | 3% Oxygen Desaturation Index | -35.361 | 19.27 | 18.441 | 0.0738 | 0.0825 | 0.4263 |
| Left Hippocampus | 3% Oxygen Desaturation Index | -17.722 | 14.429 | 16.994 | 0.2261 | 0.3204 | 0.6207 |
| Left Thalamus | 3% Oxygen Desaturation Index | 17.677 | 23.519 | 20.949 | 0.4564 | 0.4175 | 0.709 |
| Occipital Fusiform Gyrus | 3% Oxygen Desaturation Index | -53.306 | 16.354 | 26.843 | 0.0022 | 0.0739 | 0.4263 |
| Parahippocampal Gyrus anterior division | 3% Oxygen Desaturation Index | -24.78 | 16.71 | 18.715 | 0.1453 | 0.2135 | 0.5547 |
| Parahippocampal Gyrus posterior division | 3% Oxygen Desaturation Index | -7.022 | 12.541 | 15.742 | 0.5784 | 0.6646 | 0.7551 |
| Posterior limb of internal capsule L | 3% Oxygen Desaturation Index | 23.018 | 25.883 | 39.189 | 0.38 | 0.5692 | 0.7551 |
| Posterior limb of internal capsule R | 3% Oxygen Desaturation Index | -24.327 | 19.262 | 16.119 | 0.2137 | 0.1607 | 0.4982 |
| Precuneous Cortex | 3% Oxygen Desaturation Index | -23.748 | 13.767 | 11.69 | 0.0884 | 0.0685 | 0.4263 |
| Right Amygdala | 3% Oxygen Desaturation Index | -6.411 | 21.968 | 20.797 | 0.7718 | 0.7639 | 0.8031 |
| Right Hippocampus | 3% Oxygen Desaturation Index | -60.956 | 21.046 | 19.949 | 0.0049 | 0.0116 | 0.3593 |
| Right Thalamus | 3% Oxygen Desaturation Index | 4.639 | 20.301 | 20.917 | 0.8204 | 0.8288 | 0.8288 |
| Splenium of corpus callosum | 3% Oxygen Desaturation Index | -19.747 | 24.209 | 24.43 | 0.4197 | 0.4368 | 0.709 |
| Superior corona radiata L | 3% Oxygen Desaturation Index | 7.887 | 20.831 | 27.167 | 0.7068 | 0.7772 | 0.8031 |
| Superior corona radiata R | 3% Oxygen Desaturation Index | -11.539 | 23.63 | 21.914 | 0.6267 | 0.6095 | 0.7551 |
| Temporal Fusiform Cortex anterior division | 3% Oxygen Desaturation Index | -34.384 | 14.093 | 16.292 | 0.0169 | 0.0599 | 0.4263 |
| Temporal Fusiform Cortex posterior division | 3% Oxygen Desaturation Index | -17.947 | 13.22 | 16.304 | 0.1819 | 0.2957 | 0.6207 |
| Temporal Occipital Fusiform Cortex | 3% Oxygen Desaturation Index | -11.634 | 13.544 | 12.24 | 0.3951 | 0.3631 | 0.6621 |
| Uncinate fasciculus L | 3% Oxygen Desaturation Index | -69.092 | 20.992 | 30.928 | 0.0015 | 0.0485 | 0.4263 |
| Uncinate fasciculus R | 3% Oxygen Desaturation Index | -14.086 | 17.396 | 19.242 | 0.4205 | 0.4803 | 0.709 |
| Anterior limb of internal capsule L | Apnea-Hypopnea Index (AHI) | 18.037 | 27.063 | 31.176 | 0.509 | 0.5745 | 0.6596 |
| Anterior limb of internal capsule R | Apnea-Hypopnea Index (AHI) | -24.031 | 17.275 | 17.24 | 0.172 | 0.1909 | 0.4242 |
| Body of corpus callosum | Apnea-Hypopnea Index (AHI) | -11.906 | 18.191 | 14.955 | 0.5147 | 0.4428 | 0.6596 |
| Cingulate Gyrus anterior division | Apnea-Hypopnea Index (AHI) | -29.633 | 19.769 | 23.197 | 0.1416 | 0.2277 | 0.4705 |
| Cingulate Gyrus posterior division | Apnea-Hypopnea Index (AHI) | -19.445 | 17.971 | 13.365 | 0.2855 | 0.1736 | 0.4242 |
| Cingulum cingulate gyrus L | Apnea-Hypopnea Index (AHI) | -12.36 | 20.905 | 18.081 | 0.5576 | 0.5083 | 0.6596 |
| Cingulum cingulate gyrus R | Apnea-Hypopnea Index (AHI) | -21.484 | 15.415 | 11.102 | 0.1673 | 0.0791 | 0.3503 |
| Cingulum hippocampus L | Apnea-Hypopnea Index (AHI) | -35.212 | 23.923 | 25.309 | 0.1489 | 0.1916 | 0.4242 |
| Cingulum hippocampus R | Apnea-Hypopnea Index (AHI) | -11.825 | 23.35 | 19.676 | 0.6152 | 0.56 | 0.6596 |
| Genu of corpus callosum | Apnea-Hypopnea Index (AHI) | -16.777 | 26.368 | 24.453 | 0.5282 | 0.5068 | 0.6596 |
| Insular Cortex | Apnea-Hypopnea Index (AHI) | -26.155 | 10.722 | 15.458 | 0.0191 | 0.1187 | 0.4242 |
| Left Amygdala | Apnea-Hypopnea Index (AHI) | -35.345 | 18.714 | 17.991 | 0.0663 | 0.0752 | 0.3503 |
| Left Hippocampus | Apnea-Hypopnea Index (AHI) | -18.198 | 13.996 | 16.761 | 0.2006 | 0.3008 | 0.5485 |
| Left Thalamus | Apnea-Hypopnea Index (AHI) | 13.536 | 22.902 | 21.353 | 0.5577 | 0.5391 | 0.6596 |
| Occipital Fusiform Gyrus | Apnea-Hypopnea Index (AHI) | -54.383 | 15.637 | 25.244 | 0.0012 | 0.0542 | 0.3503 |
| Parahippocampal Gyrus anterior division | Apnea-Hypopnea Index (AHI) | -26.364 | 16.169 | 18.219 | 0.1104 | 0.1757 | 0.4242 |
| Parahippocampal Gyrus posterior division | Apnea-Hypopnea Index (AHI) | -8.008 | 12.165 | 15.032 | 0.5139 | 0.6048 | 0.6696 |
| Posterior limb of internal capsule L | Apnea-Hypopnea Index (AHI) | 16.437 | 25.145 | 37.148 | 0.5178 | 0.6667 | 0.7127 |
| Posterior limb of internal capsule R | Apnea-Hypopnea Index (AHI) | -25.591 | 18.629 | 16.428 | 0.1771 | 0.1475 | 0.4242 |
| Precuneous Cortex | Apnea-Hypopnea Index (AHI) | -23.625 | 13.335 | 11.697 | 0.0803 | 0.0684 | 0.3503 |
| Right Amygdala | Apnea-Hypopnea Index (AHI) | -13.814 | 21.213 | 19.542 | 0.5185 | 0.4943 | 0.6596 |
| Right Hippocampus | Apnea-Hypopnea Index (AHI) | -59.563 | 20.388 | 20.861 | 0.0045 | 0.0157 | 0.3503 |
| Right Thalamus | Apnea-Hypopnea Index (AHI) | -2.389 | 19.719 | 21.349 | 0.9042 | 0.9129 | 0.9129 |
| Splenium of corpus callosum | Apnea-Hypopnea Index (AHI) | -20.965 | 23.518 | 24.335 | 0.3785 | 0.4073 | 0.6596 |
| Superior corona radiata L | Apnea-Hypopnea Index (AHI) | 5.598 | 20.252 | 26.303 | 0.7836 | 0.8354 | 0.8632 |
| Superior corona radiata R | Apnea-Hypopnea Index (AHI) | -13.266 | 22.897 | 21.381 | 0.564 | 0.5476 | 0.6596 |
| Temporal Fusiform Cortex anterior division | Apnea-Hypopnea Index (AHI) | -36.947 | 13.548 | 16.084 | 0.0078 | 0.0422 | 0.3503 |
| Temporal Fusiform Cortex posterior division | Apnea-Hypopnea Index (AHI) | -19.049 | 12.772 | 15.608 | 0.1435 | 0.2478 | 0.4801 |
| Temporal Occipital Fusiform Cortex | Apnea-Hypopnea Index (AHI) | -16.975 | 13.003 | 11.166 | 0.1988 | 0.1566 | 0.4242 |
| Uncinate fasciculus L | Apnea-Hypopnea Index (AHI) | -67.743 | 20.322 | 29.741 | 0.0013 | 0.0437 | 0.3503 |
| Uncinate fasciculus R | Apnea-Hypopnea Index (AHI) | -11.789 | 16.884 | 19.328 | 0.4871 | 0.5543 | 0.6596 |
| Anterior limb of internal capsule L | Hypopnea Index | -18.271 | 25.291 | 26.963 | 0.4746 | 0.5139 | 0.59 |
| Anterior limb of internal capsule R | Hypopnea Index | -20.323 | 16.177 | 15.916 | 0.2168 | 0.2315 | 0.3287 |
| Body of corpus callosum | Hypopnea Index | -20.157 | 16.888 | 14.588 | 0.2362 | 0.1982 | 0.3073 |
| Cingulate Gyrus anterior division | Hypopnea Index | -48.903 | 17.47 | 19.671 | 0.008 | 0.0329 | 0.1276 |
| Cingulate Gyrus posterior division | Hypopnea Index | -30.305 | 16.286 | 13.471 | 0.0703 | 0.0491 | 0.1523 |
| Cingulum cingulate gyrus L | Hypopnea Index | -26.595 | 19.131 | 14.242 | 0.1723 | 0.0925 | 0.1777 |
| Cingulum cingulate gyrus R | Hypopnea Index | -24.376 | 14.316 | 12.146 | 0.0925 | 0.0736 | 0.1777 |
| Cingulum hippocampus L | Hypopnea Index | -34.378 | 22.39 | 19.97 | 0.1332 | 0.1169 | 0.1908 |
| Cingulum hippocampus R | Hypopnea Index | -19.751 | 21.848 | 16.356 | 0.3715 | 0.2559 | 0.3449 |
| Genu of corpus callosum | Hypopnea Index | -40.54 | 24.16 | 22.141 | 0.1017 | 0.098 | 0.1777 |
| Insular Cortex | Hypopnea Index | -38.254 | 8.999 | 11.538 | 0.0001 | 0.0082 | 0.1016 |
| Left Amygdala | Hypopnea Index | -22.964 | 18.083 | 18.063 | 0.2122 | 0.2333 | 0.3287 |
| Left Hippocampus | Hypopnea Index | -8.095 | 13.416 | 15.584 | 0.5497 | 0.6151 | 0.6811 |
| Left Thalamus | Hypopnea Index | 17.446 | 21.425 | 18.388 | 0.4204 | 0.3658 | 0.4726 |
| Occipital Fusiform Gyrus | Hypopnea Index | -64.309 | 13.499 | 22.357 | 0.0 | 0.0171 | 0.1016 |
| Parahippocampal Gyrus anterior division | Hypopnea Index | -38.497 | 14.499 | 14.754 | 0.0113 | 0.0268 | 0.1185 |
| Parahippocampal Gyrus posterior division | Hypopnea Index | 3.648 | 11.497 | 16.346 | 0.7527 | 0.828 | 0.828 |
| Posterior limb of internal capsule L | Hypopnea Index | -13.624 | 23.427 | 30.994 | 0.5651 | 0.6699 | 0.7087 |
| Posterior limb of internal capsule R | Hypopnea Index | -30.556 | 17.087 | 14.65 | 0.0818 | 0.0645 | 0.1777 |
| Precuneous Cortex | Hypopnea Index | -33.141 | 12.146 | 9.329 | 0.0078 | 0.0056 | 0.1016 |
| Right Amygdala | Hypopnea Index | -29.278 | 19.499 | 14.912 | 0.1412 | 0.079 | 0.1777 |
| Right Hippocampus | Hypopnea Index | -45.427 | 19.381 | 16.265 | 0.0216 | 0.0197 | 0.1016 |
| Right Thalamus | Hypopnea Index | 7.014 | 18.409 | 16.821 | 0.7053 | 0.6858 | 0.7087 |
| Splenium of corpus callosum | Hypopnea Index | -41.622 | 21.399 | 22.777 | 0.0599 | 0.0986 | 0.1777 |
| Superior corona radiata L | Hypopnea Index | -19.689 | 18.776 | 22.766 | 0.3007 | 0.4081 | 0.506 |
| Superior corona radiata R | Hypopnea Index | -30.33 | 21.163 | 16.055 | 0.1557 | 0.0893 | 0.1777 |
| Temporal Fusiform Cortex anterior division | Hypopnea Index | -42.866 | 12.332 | 14.767 | 0.0008 | 0.0163 | 0.1016 |
| Temporal Fusiform Cortex posterior division | Hypopnea Index | -10.96 | 12.256 | 14.969 | 0.3767 | 0.4814 | 0.574 |
| Temporal Occipital Fusiform Cortex | Hypopnea Index | -19.734 | 12.134 | 10.968 | 0.1118 | 0.1032 | 0.1777 |
| Uncinate fasciculus L | Hypopnea Index | -60.413 | 19.099 | 25.93 | 0.0022 | 0.043 | 0.148 |
| Uncinate fasciculus R | Hypopnea Index | -33.322 | 15.374 | 10.968 | 0.0332 | 0.013 | 0.1016 |

Sensitivity analysis using cluster-robust standard errors (CR2) to account for within-subject dependence arising from paired MRI sessions (d0 and d1).

Regression coefficients (β) are identical to the primary model, whereas standard errors and p values are adjusted using subject ID as the clustering variable.

ROI: region of interest

PSG: polysomnography

CR2: cluster-robust standard errors (type CR2)

FDR: false discovery rate
